# “A community unlike anything I've ever been a part of”: women mountain bikers’ experiences of social support

**DOI:** 10.3389/fspor.2026.1793325

**Published:** 2026-04-29

**Authors:** Isabella Gore Rivero, Kathleen S. Wilson

**Affiliations:** Department of Kinesiology, California State University Fullerton, Fullerton, CA, United States

**Keywords:** action sports, gender, mountain biking, social influence, social support, women

## Abstract

**Introduction:**

Mountain biking is a male-dominated action sport with differences in engagement reported between men and women. Motivation to participate in action sports like mountain biking is influenced by social aspects, including social support. The role of social support within action sports has received little attention and is not fully understood. One model that may guide our understanding of social support in this context is the *Thriving through Relationships* model by Feeney and Collins. In this model, social support allows individuals to take advantage of life opportunities, as well as managing challenges. The purpose of this study was to explore women mountain bikers’ experiences of social support within the mountain bike community, focusing on goal exploration, connections with other riders, and gender specific experiences.

**Methods:**

Twenty-two women mountain bikers (ages 29–61) participated in semi-structured virtual interviews lasting on average 51 min. Participants had varying mountain bike experience (beginner to expert) and were all from North America. Interviews were transcribed verbatim and thematic analysis was used to identify the main themes reflecting the women's experiences.

**Results:**

Participants described mountain biking as “a community building sport,” with two main themes and a third minor theme identified. Within the theme of the *women's community in a male dominated sport*, participants discussed three subthemes: a) the sport as male dominated with a lack of investment in women, b) the creation of the women's subcommunity, and c) the importance of giving back to the women's community. In the second theme of *finding the right support group,* three subthemes were identified: a) social support as function of the community, b) riding group considerations, and c) it's not just about mountain biking. The third minor theme was related to *outsider support and involvement*. Participants described that they may not be where they are without receiving social support within the sport and discussed how integral support has been to their mountain biking experience.

**Discussion:**

Overall, the findings emphasize the need to foster a supportive community for women mountain bikers.

## 1 Introduction

Action sports are a type of physical activity that have grown greatly in recent years, with the growth outpacing that of many traditional sports in Western nations (1). Action sports are a group of recreational activities that involve risk and high levels of physical demand where failure may lead to injury or death (2, 3). Popular action sports including skateboarding, surfing, skiing, and mountain biking have recently joined the Olympics and subsequently gained visibility and credibility (4). There are various motivators to participate in action sports including enjoyment, health, and mastery, which are also often seen in traditional sport. Additionally, action sports are motivated by risk, thrill, and excitement (5). Those who enjoy sensation seeking and risk taking tend to succeed in action sports (6). Initially, action sports emerged to counter some of the challenges and structures associated with traditional organized sports (7).

Gender expectations in action sports have formed in ways that differ from those seen in traditional sports (1). Early work in action sports described that while skateboarders rejected traditional masculinity, they still created a space that excluded and marginalized women (8). Further, action sports still appear to be predominantly male dominated with masculine norms (9, 10). These sports tend to be seen as masculine domains because of the high risk and danger associated (11). Woman action sport athletes report being valued based on masculine standards and are often perceived as taking fewer risks and being less technically capable (12, 13). As action sports are structured for men's success, women are often discouraged from participation (9). Rules and competitions are often defined by male standards, forcing women to either conform to masculinity or risk not succeeding in these sports (9, 10).

Mountain biking is an action sport that has gained popularity in recent years and has many positive health benefits including increases in psychological well-being, prevention of illness and disease, and improvements in fitness and strength (14). Much of the research on mountain biking has focused on men, as mountain biking is heavily male dominated (15). The popular media in mountain biking also tends to focus on men (16). Between 2008 and 2018, women received zero mountain biking screen time on Red Bull Media House, a popular action sports channel (17). The mountain bike industry overlooks women's insights, as they are a small percentage of mountain bike consumers. Unfortunately, this continues to discourage women from participating in mountain biking (15). Women mountain bikers have been found to disengage from published media and reject media written for and by men mountain bikers, in favor of curating their own mountain bike culture through social media (18). Encouraging women's participation in mountain biking is important, as woman see many health and well-being benefits from the sport (14, 19). Women mountain bikers have been found to achieve more significant psychological health outcomes than men, with benefits being seen to self-reliance, self-esteem, and life satisfaction (14).

A potential avenue for the encouragement of women's participation in mountain biking is through social support. Social support can be defined as any of the social resources available to an individual, either perceived or actual, from their social relationships (20). Within the *Thriving through Relationships* model of social support, there are two pathways through which support is provided (21). The source of strength pathways refers to support provided during times of adversity and hardship. The relational catalyst pathway refers to support provided for individuals to take part in life opportunities and push their comfort zones in the absence of adversity (21). Much of the research on social support has focused on source of strength support rather than relational catalyst support (21). Research has found that relational catalyst support results in positive perceptions of goal capability, and subsequent long-term thriving and well-being (22). Feeling capable of achieving a goal is associated with likelihood of having pursued the goal one year later (23). It is important for individuals to have someone who serves as a relational catalyst support, as this impacts feelings of capability and thriving over time (21).

Physical activity and sport participation have been found to be significantly impacted by social support. In college students, social support is associated with increased levels of physical activity (24). Social support has been shown to influence girls’ physical activity behaviors through the enhancement of self-efficacy, performance, and enjoyment (25). Tangible support such as providing equipment, money, and transportation also enables physical activity (25). Friends and family members are effective providers of social support when considering physical activity and sport behaviors (24, 26).

There is limited research exploring social support in mountain biking, and much of the research has focused on parental support for youth athletes. For youth mountain bikers, surfers, and skateboarders, parents and siblings are the main support providers (27). These family members mainly provide companionship support by participating in the activity together (27). Girls tend to face more barriers to accessing male-dominated action sports, and fathers often provide support through paternal co-participation (28). There is little research investigating the impact of social support on adult mountain bikers and woman mountain bikers.

Due to the lack of research on social support for women mountain bikers, this study serves to fill that gap by hearing from women regarding their experiences as minorities in the sport. The purpose of this study was to explore women mountain bikers’ experiences of relational catalyst social support. This study seeks to understand how and what types of support are received by women mountain bikers and how this influences their experiences in the mountain bike community in terms of goal exploration and connections with others. The secondary purpose of this study was to understand women mountain bikers’ perceptions of gender in the sport.

## 2 Methods

### 2.1 Participants

A total of 98 women indicated interest in participating in this study. Of the women who expressed interest, the first 22 individuals who were able to be scheduled were interviewed (See Table 1 for detailed participant characteristics). In order to participate, individuals needed to identify as a woman, be 18 years of age or older, be a mountain biker, and be willing to participate in a 60–90-min interview. Participants’ age ranged from 29 to 61 years old (*M* = 41.0, *SD* = 10.3). Women had been mountain biking for a range of one year to 36 years (*M* = 11.0 years, *SD* = 10.1). Reported mountain bike frequency ranged from once a month to five times per week (*M* = 2.4 times per week, *SD* = 1.3). Participants identified their mountain bike difficulty levels ranging from beginner/intermediate (comfortable riding green and blue trails) to expert (comfortable riding up to double black trails). All participants resided and mainly mountain biked in North America, with one participant living in Canada and the rest in the United States.

**Table 1:** Participant characteristics.

Pseudonym	Age	Years riding	Riding frequency (per week)	Location	Leadership
Olivia	37	25	3	USA	Coach
Charlotte	52	36	5	Texas	—
Haley	41	3	2	Arizona	—
Sam	41	11	3	Alberta	Coach
Alexa	33	1	1	East Coast	Group mentor
Marie	44	10	2	California	—
Anne	45	4	2.5	Ohio	Coach & board member
Megan	31	1	1.5	California	—
Rebecca	43	25	2.5	California	Coach
Caroline	60	30	3.5	California	—
Sophia	50	20	4	California	Coach
Liz	29	1	3	Oregon	—
Sandy	33	6	2.5	California	—
Victoria	61	11	0.75	Washington	Ride leader
Linda	30	4	2	Washington	—
Audrey	32	6	1	Arizona	—
Madeline	31	10	0.6	California	—
Susan	29	3	4.5	California	—
Jane	57	5	0.25	Florida	Group leader
Abigail	53	13	2.5	Alabama	—
Christine	44	6	2.5	California	Coach
Diane	44	18	3.5	Colorado	Coach

### 2.2 Research design

This study used a qualitative methodology to hear the voices of women mountain bikers. The study was guided by a relativist ontological and subjectivist epistemological position. This means that the findings from this study are based on the perspectives of the participants’ own experiences and through interactions with the researchers. In relation to this study, Gore Rivero acknowledges the impact of her background on how she engaged with and interpreted the data as lead researcher. Her views and understanding of mountain biking and women mountain bikers have been shaped by her experiences as a woman mountain biker herself over the past ten years. She was taught to mountain bike by her father and did not mountain bike with many other women for the first few years of learning the sport. During this time, Gore Rivero had a narrow view of what women mountain bikers could look like, with her only examples being beginner significant others of men who were serious mountain bikers. A few years after beginning, she was introduced to a few other intermediate mountain bikers and her perspective shifted to realize that women were just as capable as men in the sport. Since then, she has engaged in various women's only mountain bike events and groups and has interacted with many other women within the sport. Reflecting on these experiences, Gore Rivero acknowledges that both her time as a mountain biker riding predominantly with men and her time riding in women's only spaces have created a distinct view of the mountain bike community. This experience shaped the development of the research question and the interview questions. The interview and theme generation were led by Gore Rivero, which leads to the influence of her background, experiences, and biases as a women mountain biker affecting the findings. This experience as an insider in the mountain biking community may have assisted with developing rapport (e.g., understanding terms used by the women).While many of the participants discussed backgrounds that aligned with hers, Gore Rivero made an attempt to consider all the information gathered in interviews equally, without regard to whether or not it matched her experiences. Although, she may have viewed the women's community positively as she identifies as an active member within this community. Wilson is a woman with no experience with mountain biking or action sports. Wilson served as a critical friend throughout the process from research design through analysis and asking questions and clarifying from an outsider perspective.

### 2.3 Procedures

Institutional review board (IRB) approval was obtained prior to the beginning of participant recruitment. Recruitment consisted of displaying posters at mountain bike shops and sharing the study information and virtual posters in women's mountain biking social media pages. Interested individuals filled out a Qualtrics survey indicating their interest and verifying that they met the participation criteria. The researchers then reached out to interested individuals to schedule an interview session. Participants were given the option to meet in-person at a mutually convenient location or virtually over Zoom. All participants opted to meet over Zoom. Prior to the interview, participants were asked to review the consent form and agree to participate in the interview and to be audio and video recorded. Interviews lasted 51 min on average and ranged from 31 to 68 min. Interviews followed a semi-structured format, asking about women's experiences in mountain biking, types of support, and outcomes of receiving support (see details below). The semi-structured format allowed participants to openly share what they felt was relevant and allowed the researcher to gain a deeper understanding of the novel research question. All the interviews were conducted by Gore Rivero during the summer of 2024. Participants received no compensation or incentive for participating in the study. Data was transcribed verbatim using Dropbox transcripts to assist with the transcription. These transcripts were reviewed for accuracy by Gore Rivero who also removed all potentially identifying information. Once a preliminary analysis had taken place, participants were contacted regarding the results. Participants were sent a brief description of the themes and were given the opportunity to discuss the results with the researcher, either through email or in another Zoom interview. A follow-up email was sent to participants who had not shared their feedback two weeks after the initial request for feedback. Of the 22 participants, 15 responded with their feedback on the preliminary findings.

### 2.4 Interview guide

A semi-structured interview guide was developed prior to beginning interviews (see Supplementary Materials for the full interview guide). This guide was tested in two pilot interviews and adjusted based on feedback. There were five sections of the interview guide after introductions were made and consent was confirmed: demographic information, introduction, social support, gender, and wrap-up. The demographic information collected included age, race/ethnicity, mountain bike frequency, and types/difficulty of mountain bike trails ridden. In the introduction section, participants were asked broad questions about their relationship with mountain biking and how they got started in order to build rapport and help the participants feel comfortable. The social support section defined social support and relational catalyst support and then asked questions about received support, missing support, impacts of support, and goals/areas for growth. In the gender section, participants were asked about their experiences as a woman in the mountain bike community. Finally, the wrap-up concluded the interview by asking if participants had anything else to share and explaining that the researcher would contact the participants with the findings.

### 2.5 Analysis

Reflexive thematic analysis was used to interpret the interviews (29, 30). This is a six-step process, with the goal of drawing out a few main themes from the data. The first step of thematic analysis was familiarization with the interviews. Within this step, the researcher immersed herself with the data through transcription of the interviews and repeated reading of the transcripts with note taking. The second step was initial coding of the transcripts, where the researcher began to produce codes from the data. Codes were used to organize the data into meaningful groups. Nvivo software was used during the coding process. Step three begins the process of generating themes. In this phase, the identified codes were sorted into broader groups of themes through seeking a common meaning. In step four, researchers reviewed the themes. The themes were further analyzed in this phase to ensure that the data within each theme was clearly identifiable. The fifth step of thematic analysis is defining and naming the themes. The final step involves producing the report, where the results of thematic analysis are translated into a written format. Throughout the process of analysis, both researchers had regular discussions of the findings contributed to the reflexive nature of the thematic analysis and helped develop high quality interpretations of the data (31). These discussions focused on the themes that were developed initially by Gore Rivero with Wilson asking clarifying questions about each theme and its distinction. Discussions surrounded how themes and sub-themes were similar and different as well as and how the themes fit with the Thriving with Relationships Model. Additionally, the participants provided feedback regarding the themes in terms of how their own experiences fit within the themes, which was used to help refine the findings. This feedback did not result in significant changes to the findings but helped provide a deeper understanding of the participants’ experiences and highlighted the importance of certain findings.

### 2.6 Quality criteria

A few criteria can be considered to demonstrate the quality of this study, based on the criteria of quality qualitative research suggested by Tracey (32). This includes eight criteria for quality (worthy topic, rich rigor, sincerity, credibility, resonance, significant contribution, ethical, and meaningful coherence), of which three will be discussed in regard to this study (32). This study explored a worthy topic, in that women's experiences with social support in mountain biking are relevant to current increasing participation in the sport and the need to support women's participation (32). Sincerity in this study is demonstrated through reflexivity regarding the researchers’ biases and values, as well as through transparency of the methods used (32). Finally, credibility is shown through participant feedback on preliminary findings, allowing additional context to the findings (32). As well as, efforts were made to convey information suggested as valuable in the reporting guidelines by Braun and Clark (31).

## 3 Results

Several themes emerged within an overarching idea of a sense of support and connectedness within the mountain biking community as a whole. The mountain biking community was repeatedly described as supportive and as a welcoming place, even when negative scenarios were described. The first theme examined the development and growth of the women's mountain bike community within the male dominated sport. The second examined the role of social support within mountain biking as a community support network. A third minor theme of outsider support and involvement was identified.

### 3.1 Theme 1: women's community within a male dominated sport

#### 3.1.1 Subtheme 1.1: male dominated sport with lack of investment in women

Mountain biking, like other action sports, is heavily male dominated. Women report that “there's not a lot of other women…it's noticeable when another woman passes you” (Audrey, Experience (EX) 6 years). Organized events like co-ed rides are “always 90% men, it's never 50/50, it's never majority women” (Olivia, EX 25 years). This contributes to women feeling intimidated when beginning the sport: “I would say just in terms of like when you're first starting, especially if like it can be kind of intimidating, breaking into mountain biking, because it feels like such a male dominant sport” (Megan, EX 1 years).

Whether due to the lack of women mountain bikers, or possibly as a contributing factor to the gender imbalance, there has been a lack of investment in women from the mountain bike industry. Many women brought up that “it's super annoying not to have gear that's designed for women” and at bike shops “there are just way fewer women's clothes to pick from” (Sam, EX 11 years; Anne, EX 4 years). Additionally, women's gear and clothing that is available is often not inclusive for all types of bodies. Racing is another area which lacks proper investment in women, and often “the amount of women that are racing is so few they lump [women] all into one category no matter your age group” (Christine, EX 6 years).

With women being a minority in mountain biking, participants reported experiencing negativity and stereotyping within the sport. A commonly reported experience was receiving unsolicited advice from men riders, which was often believed to be targeted only at women riders: “You didn't stop any of the other men that were riding. Like you didn't seem concerned for them in any way, shape, or form, but me, the only gal out here today, you did decide to share your wisdom with” (Alexa, EX 1 years). At bike shops, women face the stereotype that “she doesn't know what she's talking about. She doesn't know what she's actually looking for. What she's saying is happening is not actually happening” (Rebecca, EX 25 years). Stereotyping and negativity also occur on social media where “the comments and things are just horrible sometimes” (Charlotte, EX 36 years).

When riding with men, women often have to “show the group of guys that you can do it or you can hang with them” in order to break the stereotype that “you're not as strong or you're not as willing to do something” (Rebecca, EX 25 years). When receiving this negativity, some women explained that they do not hold these comments against men specifically, and that these men are “biproducts of their environment” (Caroline, EX 30 years). Finally, a few women reported experiences where men assumed they wanted a relationship simply because they were a woman mountain biking: “I'm not going to give you my phone number… I don't want to find a boyfriend or anything like that. Like, I just want to ride” (Sam, EX 11 years).

#### 3.1.2 Subtheme 1.2: creation of women's community within male dominated sport

The women's mountain bike community had a profound influence on many of the participants, with one saying she “wouldn't be riding the stuff I ride today if it wasn't for the women in this community” (Christine, EX 6 years) The women's community was described as “a safer space in a sense where I feel like I cannot be judged” (Megan, EX 1 years). This community also was explained to be “extra supportive because it's such a male dominated sport” (Diane, EX 4 years). Within mountain biking, women's spaces created an environment that was “less intimidating and more encouraging” (Megan, EX 1 years). One participant (Jane, EX 5 years) said the following when describing her experiences in the women's community:

It is a community unlike anything I've ever been a part of because there's such diversity and acceptance, no matter age, no matter culture, sexual orientation, belief system. It's when we're all out on the trail we're all the same it doesn't matter, we're all the same. It's just this incredible bonding connection amongst us women and I have yet to really meet anybody I don't like or that I don't get along with.

When discussing the women's mountain bike community and women specific events, one participant perceived that these spaces also appeared to be inclusive for non-binary and gender nonconforming individuals in addition to those who identify as women.

When asked about who influenced their participation in mountain biking, many women discussed other women in the mountain biking community as role models: “The {location} group rides are led by this lady who's like probably in her 50s and she's just so badass. Like she's a really good role model” (Susan, EX 3 years). Role models are important for women mountain bikers to have because “it's more motivating [to] see a female do something to be like, oh, yeah, I identify with that” (Rebecca, EX 25 years). Due to the fact that there are fewer women out on the trails and some women struggle to find others to ride with, social media and “seeing folks do it on Instagram” allows for women to see themselves represented in mountain biking (Alexa, EX 1 years).

#### 3.1.3 Subtheme 1.3: giving back to the women's community

When asked about the support they receive, many women also brought into conversation the support that they give back to the community in order to keep it growing. One participant talked about how she as a youth mountain bike coach tries to “build more girls on our team, because I think it's really important as they grow, to find a sport that makes them feel empowered. And I think the sport really gives them that opportunity” (Sophia, EX 20 years). Often, more experienced riders felt that they were able to “give back more to the ride groups that [they are] riding with or the people [they are] coaching” as they grow within the sport (Sam, EX 11 years).

Possibly in response to women giving back and taking on leadership in the sport, the women's mountain bike community is described as having grown in recent years. One participant explained that “in previous years the local women's community wasn't as strong, but this year it's really kicked off” (Christine, EX 6 years). Additionally, the community as a whole is described as beginning to shift perspective towards “the acceptance of more women on bikes, more women in any sports, truly” (Victoria, EX 11 years).

### 3.2 Theme 2: finding the right support group

#### 3.2.1 Subtheme 2.1: support as a function of the community

In this study, social support was viewed through the *Thriving through Relationships* model of relational catalyst social support (21) and participants were provided a definition about this specific type of support and asked if they receive it. Participants discussed their drive to improve, creating the need for relational catalyst support: “I'm a competitor by nature. I love to compete. I love to be better. I don't necessarily have to compete against anybody else. It's competing against me” (Jane, EX 5 years). Many women discussed wanting to “prove to [themselves] that [they] can do it” (Sandy, EX 6 years). One participant described the “solid hit of dopamine when you accomplish something that's hard” and how that can be a “big motivator in and of itself” (Linda, EX 4 years).

Within women mountain bikers, support often exists from a network or community of support providers, rather than one individual support provider. Social support was described as coming from many different individuals, who almost always were also a part of the mountain bike community; this included partners, friends, riding group members, family members, and strangers on the trails. For example, one participant explains that “[her partner] is [her] main riding buddy at this point” (Diane, EX 4 years). For others, they have a combination of support depending on who they ride with:

I probably do, like, one of these organized rides once a week. And then another time during the week I ride with like a friend or a couple of other people, just like a smaller group outside of the organized ride. And then I will ride alone (Linda, EX 4 years).

Additionally, having a regular group to go ride with was emphasized by many participants: “I have built like kind of the small group of girlfriends that also recently started learning” (Sandy, EX 6 years).

#### 3.2.2 Subtheme 2.2: support network considerations (gender, skill, push, etc.)

When discussing support networks in mountain biking, participants emphasized the importance of skill considerations, gender, types of support, and a sense of acceptance. Skill considerations discussed include having someone who knows your ability, benefits of riding with more skilled riders, and benefits of having someone with your same skill level. Having someone who can tell you “this is within your competencies, you have the skills that you need to do this” was viewed as very useful (Sam, EX 11 years). Many participants preferred riding with “friends who are better than [them]” because it made the “feel like everything is possible…that's super, super motivating” (Olivia, EX 25 years).

Another important consideration of women's mountain biking support is gender. Many women discussed differences in support from men vs. from other women. Men were often described as competing with each other and “when there are guys in the picture it's just all about like speed and kind of like going the fastest or the hardest or like jumping off the tallest thing that you can do” (Linda, EX 4 years). On the other hand, riding with other women was described as “much more supportive and honest and able to be vulnerable and willing to try stuff, but in like a more controlled manner” (Sam, EX 11 years). In general, participants preferred riding with other women as they were more thoughtful, although sometimes this meant women pushed their comfort zones less due to being overly cautious.

The types of support varied among participants, however talking through features of the rides, watching each other, and having accountability were frequently discussed. One participant explained that in her riding group everyone is competitive with each other, but if “someone knows a technique we don't know or I don't know then… there's really open dialogue and, like, how do you do that?” (Liz, EX 1 years). Along with having an open dialogue, participants often brought up the value of “watching other people do it… [and seeing] they kind of went up here… and then using the stuff [they] already know to apply to that” (Caroline, EX 30 years). Accountability was another benefit of support groups, with one participant explaining that she “will always show up if someone is expecting [her]” (Olivia, EX 25 years).

The mountain biking community and individual riding groups were repeatedly described as accepting and encouraging. More experienced riders “are always trying to encourage everybody. So even though they're faster, they're always waiting for everyone and encouraging new people to come too” (Charlotte, EX 36 years). Encouragement and support are provided in all scenarios: “They’re cheering me on even if I don't successfully complete something and just the support of it's okay to fail” (Christine, EX 6 years). A common theme of not wanting to push too hard or get injured arose from participants. For these individuals who did not want to risk injury and often avoid difficult features, they discussed how they “don't feel any shame in doing that” and being “surrounded by people who haven't made [them] feel bad about doing that” (Olivia, EX 25 years). A supportive group of riders is described as individuals who “honor how [you] feel. They honor the decision that [you] make to do it or not to do it” (Jane, EX 5 years).

#### 3.2.3 Subtheme 2.3: It’s not just about biking & social aspects

The social aspect of riding with others was important to many participants. Rides were described as not just about biking; they are also an opportunity to talk about life and check in with others:

I feel like when I go with my just girlfriends, it feels like it's like we're riding because we love to ride, but it's also social hour in a way. Like we also just want to be social and just enjoy each other's company, which is maybe why we're more willing to like slow down sometimes or encourage each other to do hard things and like all of that (Linda, EX 4 years).

One participant explained that she “actually really [does] like the social aspect of it, which normally [she's] not a social person, but [she] enjoys the mountain bike community” (Susan, EX 3 years).

For many participants, relationships that developed and the support received in mountain biking extended to their life outside of riding. One participant described her group of riding buddies as her closest friends: “they're my support circle. They're my people. … I feel so lucky to have them” (Jane, EX 5 years). Another participant explained that her friends have “graduated past just biking friends” and “have become a more integral part of my daily life” (Alexa, EX 1 years).

### 3.3 Theme 3: outsider/family support & involvement

Acceptance and encouragement were also seen as coming from family members and significant others who did not mountain bike. These outsiders accepted that mountain biking was an important aspect of the participants’ life and supported them in various ways. One participant explained that much of her social support came from her partner who “doesn't particularly care about mountain biking, he comes along with me because I won't ride alone, and it makes me happy and I'm very fortunate” (Olivia, EX 25 years). For other participants, their partners were supportive of them learning to ride and they discussed how they could not “spend as much time on [their] bike… without [their partner]” (Alexa, EX 1 years).

For those who had partners who also mountain biked, it was often a way for them to connect with their partners: “I started mountain biking because I wanted to spend more time with my husband” (Jane, EX 5 years). Mountain biking was also something shared among families, with participants riding with their parents, siblings, and children.

Overall, the results indicate that social support provision is a function of the community, rather than being provided from one key support partner. Many of the women reported that they would not be where they are in mountain biking without the support they receive, and that support is an integral part of their mountain biking experience. These findings highlight the importance of fostering a supportive community for women mountain biking.

## 4 Discussion

This study examined women's mountain bikers experience with social support within the male dominated sport. The mountain bike community as a whole was frequently described as supportive and welcoming. Women reported the bulk of social support being provided by other mountain bikers, and often other women mountain bikers. This support was described for some as the reason they have stuck with the sport, which aligns with previous research finding that social support is associated with both increased levels of physical activity participation (24) and increased self-efficacy, performance, and enjoyment when participating (25). When considering a mountain bike support network, participants in this study emphasized the importance of gender. Women often felt more supported by other women and felt that riding with men was more competitive. This finding relates to a previous finding that women mountain bikers tend to prefer achieving personal goals over competition with each other, and that men and women mountain bikers are motivated to take risks in different ways (33).

Social support in this study was viewed through the *Thriving through Relationships* model, specifically aiming to understand relational catalyst support for mountain bikers (21). As mountain biking is a sport in which high risk is involved, participants described that relational catalyst support to push comfort zones and challenge oneself was a necessary component of being a mountain biker. This aligns with previous research finding that mountain bikers tend to move out of their comfort zone and frequently display thrill-seeking behaviors (34). Many of the examples that were described by participants also seemed to fit within the specific components of the relational catalyst aspect of social support (21). For example, participants discussed how riding with other mountain bikers pushed them to try more challenging features or supported them in competing against themselves may reflect the component of nurturing a desire to seize the opportunity to grow in the sport. Much of the assistance described within the women's mountain biking community when they are giving back as coaches or leading women's only events may reflect facilitating the engagement through instrumental and informational assistance. The emphasis on the value of the ‘community’ may reflect that secure base that these women need to grow within the support. While much of the research using the Thriving with Relationships model focuses on one person providing the support (e.g. 22), much of the discussion in this study focused on the support from the community of women mountain bikers. This suggests that the Thriving with Relationships model may also be beneficial for understanding the social support within groups.

Throughout this study, support was often described as being a function of the community, with support being provided from close riding buddies, group ride members, or even strangers on the trail. This function of the community appears to reflect a common social identity, where individuals’ identification with a group influences their behavior and interactions with others (35). The participants appeared to identify as a mountain biker, specifically a women mountain biker. This identity may have contributed to their willingness to help out other mountain bikers, such as participants feeling the need to give back and help out other women riders. Similarly, previous identity research has found that greater amounts of social support exist when a recipient and provider share a social identity (36). This perception of a community or social identity aligns with previous studies which discuss the culture of mountain biking as involving feelings of togetherness and inclusion with other riders (19, 37).

Beyond having a support system within mountain biking, the social aspect of the sport was important to many participants. For example, some women discussed how friends met through mountain biking became their closest friends in life. Past research has similarly found that many mountain bikers find the social aspects of mountain biking very important to their participation and prefer to mountain bike with others over riding alone (15). Additionally, when looking at action sport athletes including mountain bikers, climbers, and whitewater rafters, the social group and sense of community are reported as a main motivation for participation (38). When specifically looking at women, one study has found that women report feeling more of a social aspect when riding with other women rather than with men (39). These past findings align with what women in this study discussed regarding feeling like riding with other women was more social than riding with men and that they created strong friendships through the sport.

A key finding within the discussion of community in this study was the depth of friendships created through sport. This aligns with previous research finding that there is a large focus on establishing friendship circles in mountain biking, and many mountain bikers report having mostly friends who also mountain bike (19). Additionally, participants in another study reported mountain bike riding groups as developing into close-knit friend groups that met outside of the context of the sport (38). For women specifically, past research has found that women's only mountain biking groups provide a space for forming relationships (40), which was also found in this study.

In addition to exploring social support in mountain biking, this study also aimed to explore women mountain bikers’ experiences in the male dominated sport. Women in this study frequently discussed the fact that there are far fewer women who mountain bike than men, which aligns with previous research on demographics in mountain biking (14, 41). Previous research has found that one of the main constraints to women mountain biking is the lack of other women who participate in the sport (42). Within the discussion of women's underrepresentation in the sport, participants often brought up the idea that there is a lack of structural investment in the sport. The main topic discussed within this lack of investment was the lack of women's specific mountain bike gear available for purchase. Previous literature on women mountain bikers has highlighted similar frustration with the lack of women's mountain biking gear and the fact that most women's specific mountain bike gear is available only in pink or other “girly” colors (37, 40).

As a minority in the sport, women discussed negativity and stereotyping being a part of their experience in mountain biking. As a result of existing stereotypes that women are inferior to men mountain bikers, women in this study and previous studies describe feeling the need to prove themselves when riding with men and are praised for being able to keep up with the guys (39). One of the most common experiences shared among participants was unsolicited advice and a feeling of being talked down to by men, both on the trails and at mountain bike shops. This finding is mirrored in an action sport study in which women surfers describe uninvited coaching from men they surf with who perceive all women as less skilled than men (13). These additional efforts of men to try and help the women mountain bikers may be reflective of benevolent sexism or paternalism (43), in that the men viewed the women as less capable and in need of help. This type of sexism may lead to self-doubt or decreased feelings of competence and potentially have impacts for women's performance. While the women mountain bikers talked about the value of social support from the right group, it should be noted that this unsolicited advice or help has potential negative impact if perceived to be coming from the stereotype of women as being inferior.

While there were not specific questions aimed at understanding support provision, women in this study frequently brought up the need they feel to support other women and to give back to the mountain bike community. This finding relates to research done on professional women athletes which discusses the pressure women feel to serve as a source of inspiration for other girls and women, and this inspirational labor largely tends to be unpaid (44). Similarly, societal expectations cause many women to take part in unpaid, invisible, and emotion-based work under the assumption that women need to be caregivers and provide for others (45). This idea was prevalent in participants’ discussions of feeling the need to give back to the women's mountain bike community by becoming involved in leadership or taking on a support role for newer riders. While there are numerous possible interpretations for why women felt the need to give back, one of the main drivers seemed to be the effort to sustain the community. Participants talked about wanting to provide support to other women that they did not receive themselves, while others talked about how they wanted to continue to provide the support to others that was so valuable to themselves. These efforts by the women to sustain this community may reflect the social identity perspective where people act to maintain their social group (46) or it may be reflective of pressures or expectations that women are expected to support each other.

This study found that for many women, the women's mountain bike community was integral to their experience, and the community was described as more welcoming and supportive. Within research on action sports, this emphasis on women's only spaces has been frequently discussed, as well as the importance of women role models (37, 39, 40). In addition to in-person women's only spaces, participants in this study also mentioned the importance of representation in social media and the use of platforms such as Instagram to connect with other women riders and see themselves represented in the sport. This aligns with a recent study which found that women mountain bikers tend to feel disengaged from much of the mountain bike media catering to and focusing on men, but make an effort to curate their own alternative women's mountain bike culture using social media (18).

There were various strengths to this novel study. This study began to fill the gap in the research surrounding women's experiences in mountain biking and to our knowledge it is the first qualitative study to focus on women's social support in mountain biking. The sample was varied in terms of years of experience, age, riding difficulty level, frequency, and represented many areas in North America.

While this study used the Thriving through Relationships model (21) to guide the main purpose, one limitation of this study is that this model does not address potential gender experiences specifically. Another main limitation of this study was the recruitment of participants. Participants were recruited predominantly through social media groups related to women's mountain biking. This may have resulted in recruitment of women who had artificially higher involvement in the community than those who are not in these groups. A large portion of the women interviewed were involved in leadership in the mountain bike community, which may not be an accurate representative of all women mountain bikers. Another limitation is the lack of racial and ethnic diversity, with the majority of women interviewed identifying as white. This study also only investigated those who identify as women and did not take into account the experiences of non-binary or other gender minority mountain bikers. While one participant perceived these spaces as ‘inclusive’ to gender identities, more research is needed from those groups.

Future research in this area could further investigate the internal pressure women mountain bikers feel to give back to their community. Research could also attempt to ask similar questions to women who are not a part of social media groups for women mountain bikers to understand differences in support. Similarly, future research could investigate the experiences of women who dropped out of the sport to understand differences in support that may lead to drop out. Previous research has found many similarities in gendered experiences of mountain bikers and other action sport athletes. Future studies could attempt to understand how the findings of this study apply to other male dominated action sports such as skiing or surfing. Finally, future studies could target non-white women, as well as non-binary mountain bikers to understand their experiences in the sport.

The purpose of this study was to explore women mountain bikers’ experiences of social support within the mountain bike community, focusing on goal exploration, connections with other riders, and gender specific experiences. Interviews with women mountain bikers uncovered the importance of community in providing support within the sport, as well as shed light on the unique experiences of women in the male dominated sport. Several practical applications can be taken from the results of this study. Support for women and women's only spaces is important for women's development and inclusion in mountain biking. This also includes the development of equipment and gear designed for women. Further, potential efforts could be made to address the stereotypes of women mountain bikers through sharing stories and communicating about the successful women riders. This may potentially reduce the negativity experienced from other riders. Additionally, mountain bike organizations should ensure women's voices are included in leadership positions to share the unique experiences of women.

## Data Availability

The datasets presented in this article are not readily available. To protect the confidentiality of our participants, data is not available to be shared. Upon request, further quotes from participants within each theme may be shared. Requests to access the datasets should be directed to Kathleen Wilson, kswilson@fullerton.edu.
