# Supplementary material for: “A community unlike anything I've ever been a part of”: women mountain bikers’ experiences of social support
Source: Front Sports Act Living. 2026 Apr 29;8:1793325. doi: 10.3389/fspor.2026.1793325 (PMC13167974; doi:10.3389/fspor.2026.1793325)
Supplement: Supplementary file 1 [file Supplementaryfile1.docx]

Supplementary Material: Interview Guide

Thank you for taking the time to meet with me today. My name is NAME, and today we’re going to be talking about your experiences with social support in mountain biking. I’ll start by introducing myself, I am currently getting my Master’s in kinesiology with an emphasis in sport psychology. I’m also an avid mountain biker and advocate for women in sports. I am doing this research project to prepare for my thesis, which will also look into women’s experiences in mountain biking.

**Demographics**

We’re going to move on to the main interview now. In this interview, I have some questions prepared for you, but I may ask follow ups based on what you say. Please feel free to ask for clarification or examples at any point. Sounds good?

I am going to start off my collecting some demographic information.

- How old are you?
- What is your race or ethnicity?
- How long have you been mountain biking?
- About how frequently do you mountain bike?
- What type of trails do you tend to ride? (type and difficulty, where)
- How did you hear about this study?

**Introduction**

- I want to start by learning more about your relationship with mountain biking. Tell me about why you mountain bike and some things that you like about it.
- How did you get into mountain biking? (who introduced you, how were you supported, etc.)
  - Did someone introduce you to the sport?
  - What sparked interest?
  - What did you think about it the first time you mountain biked?
- Tell me about who has influenced your participation in mountain biking. (can be one person or more)
  - Who has been involved in your mountain biking experience?
  - Fellow mountain bikers AND anyone outside of the sport.
  - Is there anyone who has made being a mountain biker easier?

**Social Support**

This study is looking at social support specifically. I want to make sure you understand what I am referring to when I say social support, so for the purposes of this study, social support is the resources that you have available to you from your social relationships (friends, family, etc.). This can come in many forms, for example someone encouraging you, providing financial support, or teaching you something. Does that make sense?

- Tell me about the social support that you receive in mountain biking.
  - Who provides it?
  - What types of support?
  - How does it impact your experiences?
- Is there any support that you feel you are missing regarding mountain biking?

There is a specific type of social support that I am looking at in this study. It’s called relational catalyst support, but you don’t need to remember that. This type of support is aimed at helping you grow and push outside of your comfort zone. This support can be things like helping you recognize opportunities in your life or encouraging you to try new things. Does that make sense?

- Do you have someone who provides that kind of support to you in mountain biking?
- IF YES: tell me about them and what they do for you.
  - Please tell me an example of how they support you that made a big difference for you.
  - What outcomes does their support have on you? (e.g., confidence, motivation, comfort, etc.)
  - What is your relationship with them? (e.g., mountain biker or not, significant other, friend, family, etc.) Where do you interact with them?
  - Gender?
  - How long have you known them? How long have they been a support person for you?
- IF NO: have you ever had someone like that in mountain biking?
  - - Yes – above questions.
    - No -- Tell me about how others impact your mountain biking.
- Have you ever had a time when you aren’t sure about trying something when mountain biking?
  - If yes, please share a little about this experience? How did (support partner’s name) respond? Can you provide an example of this.
  - If no, what would you do if you came up on this in the future. What could (support partner/network) do to help you?
- *How has your support in mountain biking changed from when you first started to now?

*An important aspect of the relational catalyst model is focusing on goals and exploration.

- What growth or goals have you worked towards/are you currently working towards in mountain biking?
  - If want examples: This could be something like advancing to a harder trail level, riding more often, improving technically, making more friends in the mountain biking community, etc.
- In what ways has your support partner/network helped you work towards these goals or areas for growth?
- Besides your support person/network, what motivates you to try new things and push outside of your comfort zone when mountain biking?

**Gender**

As you know, we are specifically looking at women’s experiences in mountain biking.

- Tell me about your experiences as a woman in the mountain bike community.
  - How do you think your gender has impacted your experiences as a mountain biker?
  - What would you tell another woman who is just starting to mountain bike?
- How has your support person/network impacted your experiences as a woman in mountain biking?
  - Women vs. men in support network
- Is there anything else you would like to share with me about social support or about being a woman in mountain biking?

**Wrap-Up**

I will be contacting you once we have completed all data collection and analysis to share our main findings and discuss the central themes that we see in these interviews. We will discuss our findings with you, either via email or in another brief interview if you are interested. Be sure to look out for that email in the future.

Thank you for taking the time to meet with me for this interview.
